# A systematic review of literature to evaluate the burden of physical and psychological symptoms and palliative care in patients diagnosed with idiopathic pulmonary fibrosis

**DOI:** 10.7196/AJTCCM.2019.v25i1.231

**Published:** 2019-04-12

**Authors:** M F van Jaarsveld, L Gwyther

**Affiliations:** Division of Family Medicine, School of Public Health and Family Medicine, University of Cape Town, South Africa

**Keywords:** idiopathic pulmonary fibrosis, quality of life, palliative care

## Abstract

**Background:**

Palliative care improves quality of life in patients with life-threatening illnesses, such as idiopathic pulmonary fibrosis (IPF), in a holistic manner and should be integrated early into the management of these patients.

**Objective:**

To evaluate the degree of physical and psychological symptoms in patients with IPF and the extent to which palliative care is used in patient management.

**Methods:**

Several databases were searched for studies reporting on symptom burden, quality of life or palliative interventions in patients with IPF.

**Results:**

A total of 46 articles were included in this review. Studies showed that many patients experienced dyspnoea, which improved with pulmonary rehabilitation in some cases. Fatigue and poor quality of sleep had a notable negative impact on daily life activities. Instruments evaluating anxiety and depression showed that many patients with IPF experienced mild to moderate depression and anxiety. Quality of life was shown to be negatively affected across all domains. Two studies indicated poor referral to palliative care units and one study reported positively on the use of morphine for managing breathlessness in advanced IPF.

**Conclusion:**

Patients with IPF generally experience poor quality of life. Patients are seldom referred to palliative care, even in developed countries. No data were available on the use of palliative care in developing countries. Furthermore, research on the burden of symptoms and management of these symptoms appears to be limited. Increased awareness of and research on the palliative care needs of patients with IPF are recommended, particularly in resource-limited settings such as South Africa.

## Background

Idiopathic pulmonary fibrosis (IPF) is a chronic, progressive pulmonary disease associated with a poor life expectancy, similar to lung cancer. The annual incidence is seemingly increasing, with the current prevalence in the USA estimated at 13 - 20 cases per 100 000 and seen predominantly in men older than 60 years.^[1]^ However, there is a lack of data on the prevalence and incidence of IPF in developing countries. In general, the diagnosis is delayed^[2]^ and consequently the disease is already advanced by the time of diagnosis, especially in countries with restricted access to high-resolution computed tomography imaging and pulmonary biopsies.

Current treatment focuses on the use of antifibrotic agents, which has been shown to reduce disease progression and improve lung function;^[3]^ however, no significant effect has been observed with regard to the quality of life of IPF patients. Lung transplantation remains the most effective way of treating this devastating disease and IPF is indeed the most common indication for lung transplantation in developed countries.^[4]^ However, the procedure needs to be done early in the disease trajectory and effective post-transplant care is critical. In developing countries, the disease is usually more advanced by the time of diagnosis owing to delayed diagnosis, with the lack of transplant centres, shortage of donor organs and restricted access to antifibrotic agents contributing to an already significant symptom burden and potentially low quality of life.

Palliative care is defined by the World Health Organization as ‘an approach that improves the quality of life of patients and their families facing problems associated with life-threatening illness through the prevention and relief of suffering, the early identification and impeccable assessment and treatment of pain and other physical, psychosocial and spiritual problems’.^[5]^ By definition, palliative care should be integrated early into the management of every patient diagnosed with IPF.

This systematic review therefore aimed to evaluate the degree of physical and psychological symptoms of patients with IPF, together with their reported quality of life, and to assess the extent of palliative care involved in managing these burdensome symptoms.

## Methods

The methodology of this study is based on the PRISMA Statement for Reporting Systematic Reviews and Meta-Analyses.^[6]^ Medline, PubMed and Advanced Google Scholar databases, together with reference lists of several review and research articles, were searched for studies reporting on symptom burden, quality of life or palliative care in IPF patients older than 18 years and which had been published between January 2000 and December 2016. The studies included in the review were not limited to a specific design or disease stage to allow a better overall impression of problematic symptoms from the time of diagnosis.

## Results

A total of 23 955 articles were initially identified. After screening for titles, abstracts and duplicates, 113 full articles were selected. Of these, 46 were included in our study.

Dyspnoea was evaluated mainly according to the Modified Medical Research Council (mMRC) Scale (Table 1). Patients presented at different stages of the disease; namely: newly diagnosed;^[7,8]^ consecutive follow-up,^[9–12]^ and stable disease.^[13,14]^ The studies by Kozu *et al*.^[13]^ and Ozalevli *et al*.^[14]^ showed that the mMRC scores significantly improved following 8 weeks of home-based pulmonary rehabilitation.

**Table 1:** Studies that evaluated dyspnoea according to the Modified Medical Research Council Scale

**Study**	**Year of publication**	***N***	**Disease severity**	Results (Grade(%))*
Nishiyama *et al*.^[7]^	2010	93	Consecutive newly diagnosed patients	I: 44%
				II: 40%
Kolilekas *et al*.^[8]^	2013	31	Consecutive newly diagnosed patients	I: 29%
				II: 41.9%
				III: 16.1%
Tzanakis *et al*.^[9]^	2005	25	Consecutive patients at specialist clinic	2.04 (1.1)
				Healthy controls: 0.3 (0.1)
Baddini Martinez *et al*.^[10]^	2002	30	Consecutive patients seen at specialist clinic	I: 40%
				II: 26.67%
				III: 20%
Atkins *et al*.^[11]^	2016	77	Consecutive patients identified from database	II: 38.7 %
				III: 30.7%
Manali *et al*.^[12]^	2010	25	Consecutive patients at specialist clinic	I: 34%
				II: 34%
				III: 24%
Kozu *et al*.^[13]^	2011	45	Stable disease	II: 31%
				III: 33%
				IV: 36%
				Mean: 3.08 (0.8)
				After PR: 2.5 (1.1); p<0.01
Ozalevli *et al*.^[14]^	2010	17	Stable disease	Before PR: 2.3 (1.2)
				After PR: 1.4 (1.2); p=0.003

*Results are presented as mean (SD) or a percentage.

PR = pulmonary rehabilitation.

Two studies evaluated fatigue as outcome according to the Fatigue Severity Scale (data not shown). The results indicated that fatigue had a substantial negative impact on the respondents’ daily life activities. The Pittsburgh Sleep Quality Index (total score), the Epworth Sleepiness Scale and the Functional Outcome in Sleep Questionnaire were used in a few studies to evaluate sleep (data not shown). In most cases, the results were abnormal, which suggests considerable daytime sleepiness. Table 2 presents a summary of the studies^[15–18]^ that evaluated obstructive sleep apnoea (OSA) as an outcome.

**Table 2:** Studies that evaluated obstructive sleep apnoea according to the apnoea–hypopnoea index in patients with idiopathic pulmonary fibrosis

**Study**	**Year of publication **	***N***	**Disease severity**	Results*
Mermigkis *et al*.^[15]^	2013	23	Consecutive newly diagnosed patients	Mild OSA: 26%
				Moderate to severe OSA: 52%
Mermigkis *et al*.^[16]^	2015	92	Consecutive newly diagnosed patients	Mild OSA: 20%
				Moderate to severe OSA: 65%
Lancaster *et al*.^[17]^	2009	50	Consecutive patients at specialist lung clinic	Mild OSA: 20%
				Moderate to severe OSA: 68%
Pihtili *et al*.^[18]^	2013	17	Consecutive patients	82.3% of patients experienced OSA

*According to the apnoea–hypopnoea index:

· Normal: <5 events per hour

· Mild OSA 5 - 15 events per hour

· Moderate to severe OSA: >15 events per hour

(OSA = obstructive sleep apnoea)

**Fig. 1:**
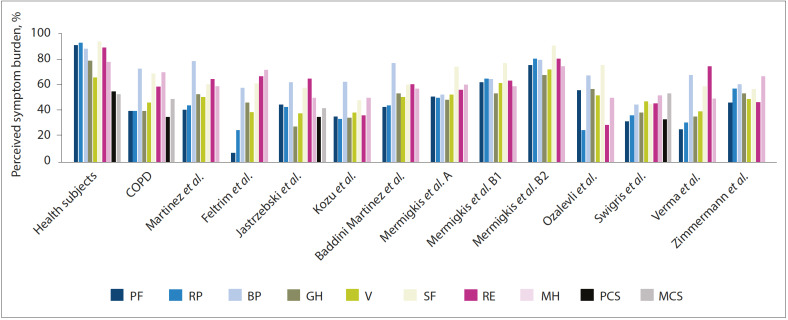
Perceived quality of life in patients with idiopathic pulmonary fibrosis, as measured with the Medical Outcomes Study Short Form 36 questionnaire. PF = physical function RP = physical role BP = bodily pain GH = general health perception V = energy/vitality SF = social function RE = emotional role MH = mental health PCS = physical component summary MCS = mental component summary

The Beck Depression Inventory and the Hospital Anxiety and Depression Scale were used to evaluate symptoms of depression and anxiety in IPF patients at different disease stages. The results of seven studies (data not shown) indicated that patients experienced mild to moderate depression and anxiety. The Medical Outcomes Study Short Form 36 (SF-36), a generic self-administered questionnaire that measures general health status across eight health domains, was used in 10 studies^[10,13,14,16,19-24]^ to evaluate quality of life in patients with IPF (Fig. 1). The scores are given as the percentage of health impairment, with a score of 0 representing ‘worst health’ and a score of 100 indicating ‘best health’. We included the results of the SF-36 completed by both healthy subjects of working age^[25]^ and patients with chronic obstructive pulmonary disease (COPD)^[26]^ for comparison in Fig. 1.

Mermigkis *et al*.^[16]^ analysed SF-36 results in IPF patients who were diagnosed with moderate to severe OSA. In Fig. 1, the results depicted at Mermigkis *et al*. A^[16]^ represent the baseline SF-36 results of the patients who had difficulty in complying with the use of continuous positive airway pressure (CPAP) therapy at night (N=18), whereas the data shown at Mermigkis *et al*. B1^[16]^ represent patients who were able to use CPAP therapy for at least 6 hours every night (N=37). The results at Mermigkis *et al*. B2^[16]^ represent the patients from B1 who were on effective CPAP therapy after 1 year. There was a statistically significant improvement in all the domains, with all p-values <0.05.

**Fig. 2:**
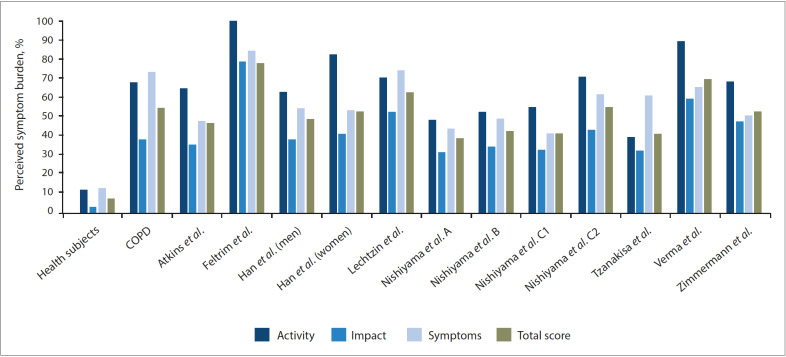
Perceived symptom burden in patients with idiopathic pulmonary fibrosis, as measured with the St. George’s Respiratory Questionnaire.

The St. George’s Respiratory Questionnaire (SGRQ) (Fig. 2) is a disease-specific instrument initially validated to assess the effect of dyspnoea on quality of life in patients diagnosed with COPD. Higher scores represent worse health status. The SGRQ has been used extensively in patients with IPF. Atkins *et al*.^[11]^ compared SGRQ scores in patients with sarcoidosis (data not shown) and patients with IPF (data shown), and found significantly worse health scores both in the activity domain (p=0.031) and in the total scores of patients diagnosed with IPF. The results of Feltrim *et al*.^[19]^ showed high SGRQ scores (poor health status), as illustrated by the graph. Patients on the waiting list for a lung transplant were included in this study. Han *et al*.^[27]^ compared quality of life between male and female patients diagnosed with IPF. They found no significant difference in the total SGRQ scores, although activity scores were better in male than in female patients. Lechtzin *et al*.^[28]^ found reduced quality of life in stable IPF patients, with most scores above 50. Nishiyama *et al*.^[29–31]^ reported on Japanese patients in three different studies. The study reported as Nishiyama *et al*. A^[29]^ in Fig. 2 included the results of 87 patients newly diagnosed with IPF, while that shown as Nishiyama *et al*. B^[30]^ included the results of 41 consecutive but stable patients. Nishiyama *et al*. C1 and C2^[31]^ show results from 28 Japanese patients, respectively before and after a 10-week outpatient pulmonary rehabilitation programme. No significant differences were found at completion of the programme.

Ten studies included in the review reported on symptoms at presentation using a direct question on the symptoms patients experienced as most bothersome (results not shown). Dyspnoea, cough and fatigue were mentioned as problematic symptoms that affected quality of life in most of these studies.

Only two studies evaluated referral to palliative care services. Lindell *et al*.^[32]^ found that only 13.7% of patients who did not qualify for lung transplantation were referred to palliative care services from a specialist lung clinic. Most of the patients died within 1 month of their referral. Rajala *et al*.^[33]^ reported on data from files of deceased patients with IPF from Finland, with no documented referrals to palliative care services. A study on the use of morphine to treat breathlessness in patients with advanced IPF showed no negative effects.^[34]^

## Discussion

The aims of this systematic review were to evaluate physical and psychological symptoms experienced by patients diagnosed with IPF and to review how palliative care contributed to the management of these burdensome symptoms. This review included 46 studies, each reporting on some aspect(s) of symptom complex or quality of life of IPF patients. However, none of the studies were specifically designed to evaluate the burden of symptoms of patients with IPF in the general population; the inclusion and exclusion criteria of studies were designed to meet the specific aims or objectives of an individual study and patients with severe symptoms or advanced disease were often excluded. This review is therefore not representative of all patients with IPF. Very few studies included in this literature review originated from developing countries, where an even higher burden of symptoms is expected among patients with IPF. Few studies reported on palliative symptom management and no studies were conducted by palliative care teams. Also, few patients from the reported studies were referred for formal palliative care and in most cases data were collected retrospectively from case files, which again do not necessarily represent the true symptom burden of a patient diagnosed with or dying from IPF. However, the combined results present an overall impression of the symptoms experienced by patients with IPF.

Dyspnoea and cough were the most common symptoms described by patients with IPF. Data from studies using the mMRC scale (Table 1) showed a significant degree of dyspnoea at the time of diagnosis throughout. Some studies demonstrated significant improvement in the mMRC scores for dyspnoea following home-based pulmonary rehabilitation,^[13,14]^ reinforcing the importance of an interdisciplinary approach in the treatment of these patients. Dyspnoea and cough correlated with the limitations in daily activity experienced by patients with IPF, as demonstrated by the SGRQ (Fig. 2). Although IPF patients experienced impairment in all the domains compared with healthy subjects, higher scores were seen in the activity domain in most of the studies, reflecting more severe limitations of activity due to dyspnoea and cough. The variation in the results across different studies represented different disease stages of patients included in the particular study.

Fatigue or ‘overwhelming exhaustion’ is frequently described by patients with IPF. In this review, studies reporting on fatigue indicated that 30 - 50% of patients experienced fatigue at the various disease stages.

Few studies reported on the quality of sleep in patients with IPF, or on the possible impact of poor-quality sleep on general wellbeing and quality of life. Most of the studies that reported on symptoms did not mention sleep quality as a major concern (data not shown), although a survey among patients with IPF in Ireland indicated poor quality of sleep in 55% of respondents.^[35]^ However, results from the Pittsburgh Sleep Quality Index (total score), Epworth Sleepiness Scale and the Functional Outcome in Sleep Questionnaire were abnormal in most cases, which points to considerable daytime sleepiness.

OSA occurs in 2 - 4% of healthy adults and is generally associated with obesity or anatomical abnormalities of the respiratory tract. The pathophysiology of OSA in patients with IPF is not clear and it is suggested that inflammation may have a critical role. The incidence of OSA in patients with IPF is high. In four studies^[15–18]^ included in this review, mild to severe OSA was found in 70 - 80% of patients with IPF. Although different diagnostic criteria were used to grade and diagnose OSA in these studies, they all consistently reported a high incidence.

Psychological distress is common in patients with advanced respiratory disease. In patients with COPD, a study by Lou *et al*.^[36]^ found the prevalence of depression and anxiety to be 35.7% and 18.3%, respectively. Holland *et al*.^[37]^ found the prevalence of anxiety to be 31% and of depression 23% in patients with interstitial lung disease. In the current review, only a few studies reported on depression and anxiety. In the four studies that presented results obtained with the Beck Depression Inventory, the mean baseline scores were above the threshold for normal throughout (data not shown), indicating mild to moderate depression. Results from three studies using the Hospital Anxiety and Depression Scale indicated depression in 12 - 24% of participants and anxiety in 25 - 38% (data not shown). A similar proportion of patients (37%) reported anxiety in the Irish survey.^[35]^ Results from the SF-36 scale (Fig. 1) demonstrated the emotional domain to be affected in nearly all the studies reviewed, indicating a negative effect on quality of life. However, except for pain, this domain was affected to a lesser degree than the others in most studies.

All the studies that reported on the SF-36 (Fig. 1) demonstrated poor health-related quality of life in the various domains compared with that of healthy controls. However, physical functioning, which examines the limitations in physical activity, and the effect of physical health on work or other daily activities (role - physical) were consistently scored lower. These findings correlate with results from the SGRQ described earlier: the activity domain, which ultimately measures the effect of disturbances in mobility and physical activity secondary to the respiratory condition, was found to be the worst affected. Patients with IPF therefore appear to experience worsening quality of life in all the various domains throughout the disease trajectory.

Despite IPF being a life-limiting disease with poor general prognosis, very few patients appear to be referred to specialised palliative care services in developed countries, where these services are readily available. Few studies reported on symptoms during the last days of life (data not shown). Reports on the treatment of these symptoms confirmed that the basic palliative care medication for treating breathlessness, anxiety and other distressing symptoms is available and safe.^[34,35]^ However, as reported by Bajwah *et al*., ^[38]^ healthcare workers, including doctors, are often not comfortable using these medications owing to limited exposure and fear of side-effects. Therefore, it would not be surprising to find that most IPF patients succumb unexpectedly in acute hospital settings in developing countries, where palliative care services are limited. Unfortunately this often occurs in emergency departments, causing great physical and emotional suffering to patients and family members.

## Conclusion

Despite several studies focusing on the various aspects of quality of life in IPF patients and the effect of disease-oriented treatment on quality of life, limited research is available on the burden of symptoms of this disease. It is also important to note that in the studies that are available, including those that were reviewed here, patients were generally selected according to specific inclusion criteria and so do not necessarily represent the typical patient living with IPF.

From the reviewed literature, it is clear that only a small percentage of patients are referred for formal palliative care. None of the reviewed publications included patients from resource-limited countries and, therefore, even less is known regarding their symptom burden and care. However, this review confirmed that patients with IPF experience poor quality of life in all the domains of daily living. Dyspnoea, cough and fatigue were identified as the most burdensome symptoms with regard to quality of life. Pulmonary rehabilitation has been shown to improve the quality of life in some patients with IPF, which confirms the importance of an interdisciplinary treatment approach, especially in resource-limited settings.
